# Modulating Matrix Metalloproteinase Activity in Obesity: Comparative Effects of Bariatric Surgery and GLP-1/GIP-Based Pharmacotherapy

**DOI:** 10.3390/jcm14217648

**Published:** 2025-10-28

**Authors:** Konrad Wiśniewski, Barbara Choromańska, Mateusz Maciejczyk, Jacek Dadan, Piotr Myśliwiec

**Affiliations:** 1 1st Department of General and Endocrine Surgery, Medical University of Bialystok, 24a M. Sklodowskiej-Curie Street, 15-276 Bialystok, Poland; 2 Department of Hygiene, Epidemiology and Ergonomics, Medical University of Bialystok, 2c A. Mickiewicza, 15-369 Białystok, Poland

**Keywords:** obesity, matrix metalloproteinases (MMPs), extracellular matrix (ECM), bariatric surgery, GLP-1 receptor agonists, dual GIP/GLP-1 receptor agonists, inflammation, metabolic remodeling, insulin resistance

## Abstract

Obesity is a multifactorial metabolic disease characterized by chronic low-grade inflammation, extracellular matrix (ECM) dysfunction, and systemic metabolic dysregulation. Matrix metalloproteinases (MMPs), especially MMP-2 and MMP-9, are key regulators of ECM remodeling and inflammation in obesity. This narrative review aimed to synthesize and critically discuss current evidence on the effects of bariatric surgery and pharmacological therapies, including GLP-1 and dual GLP-1/GIP receptor agonists, on MMP activity and metabolic outcomes. Literature from PubMed and Scopus and Web of Science (2015–2024) was analyzed, focusing on studies evaluating MMPs, inflammation, and metabolic parameters. Bariatric surgery consistently reduces MMP-9 levels and normalizes MMP-2 activity, contributing to improved ECM integrity, reduced inflammation, and enhanced insulin sensitivity. Pharmacological therapies achieve substantial weight loss and glycemic control, but evidence regarding their direct effects on MMP activity remains limited. This review highlights bariatric surgery as the most effective strategy for modulating obesity-related MMP dysregulation and emphasizes the need for further research into the mechanistic effects of modern pharmacotherapy on ECM remodeling.

## 1. Introduction

Obesity is one of the most pressing global health challenges of the 21st century, associated not only with excess body weight but also with severe comorbidities such as type 2 diabetes mellitus (T2DM), hypertension, cardiovascular disease, and several malignancies [1,2]. Beyond energy imbalance, obesity is increasingly recognized as a state of chronic low-grade inflammation that promotes metabolic dysfunction and accelerates disease progression [3,4].

In recent years, therapeutic approaches to obesity have evolved substantially, extending beyond surgical interventions to include modern non-invasive pharmacological therapies such as glucagon-like peptide-1 receptor agonists (GLP-1 RAs) and dual GLP-1/glucose-dependent insulinotropic peptide (GIP) receptor agonists [5,6,7]. These incretin-based agents achieve clinically significant and durable weight loss comparable to some bariatric procedures while simultaneously improving glycemic control and exerting anti-inflammatory effects [5,6]. Mechanistically, activation of the GLP-1 receptor modulates immune cell activity, decreases macrophage infiltration in adipose tissue, and downregulates pro-inflammatory cytokines such as TNF-α, IL-6, and CRP, thereby contributing to vascular and metabolic protection [8,9]. Moreover, by suppressing NF-κB-dependent transcription and oxidative stress, GLP-1 signaling may indirectly influence extracellular matrix (ECM) remodeling, stabilizing its structure and limiting fibrosis [4,8,9]. Although direct evidence remains limited, preliminary findings suggest that incretin-based pharmacotherapy reduces circulating MMP-2 and MMP-9 levels [8,9], highlighting its potential to restore ECM homeostasis and attenuate chronic inflammation in obesity.

ECM remodeling plays a central role in the pathophysiology of obesity. Structural and functional alterations in adipose tissue, including fibrosis, hypoxia, and dysregulated angiogenesis, are driven by imbalances in ECM homeostasis [8,9]. Matrix metalloproteinases (MMPs), a family of zinc-dependent endopeptidases, are pivotal mediators of ECM degradation and tissue remodeling [10]. Among them, MMP-2, MMP-9, and MMP-14 are particularly important due to their links with systemic inflammation [2], insulin resistance [11], and vascular complications [12].

Bariatric surgery remains the most effective treatment for obesity, leading to profound weight reduction and remission of metabolic comorbidities [13]. Recent evidence suggests that its beneficial effects may be partly mediated by modulation of MMP activity [12,13]. Meanwhile, incretin-based pharmacotherapies offer a promising less invasive alternative, though their precise impact on ECM remodeling and MMP regulation remains to be fully elucidated.

The following section synthesizes and critically discusses current evidence on the effects of bariatric surgery and incretin-based pharmacotherapy, including GLP-1 and dual GLP-1/GIP receptor agonists, on MMP activity, ECM remodeling, and metabolic outcomes, based on literature identified through a focused, but not fully systematic, search.

## 2. Materials and Methods

This narrative review was conducted to summarize and critically discuss current evidence on the effects of bariatric surgery and pharmacological therapies, including GLP-1 and dual GLP-1/GIP receptor agonists, on matrix metalloproteinase (MMP) activity and metabolic outcomes.

### 2.1. Literature Search Strategy

A focused literature search was performed in PubMed, Scopus, and Web of Science databases for articles published between January 2015 and March 2024. The following Boolean search string was applied (adapted to each database syntax): (“matrix metalloproteinase” OR “MMP”) AND (“obesity” OR “adipose tissue”) AND (“bariatric surgery” OR “gastric bypass” OR “sleeve gastrectomy” OR “metabolic surgery” OR “GLP-1 receptor agonist” OR “GLP-1/GIP agonist” OR “tirzepatide” OR “semaglutide” OR “liraglutide” OR “pharmacotherapy”).

The search was restricted to English-language publications involving human or animal studies. Reference lists of relevant reviews and clinical trials were manually screened to identify additional studies not captured by the database search.

### 2.2. Inclusion and Exclusion Criteria

Inclusion criteria:

1. Original experimental, translational, or clinical studies investigating MMP activity, expression, or regulation in the context of obesity or its treatment.
2. Studies evaluating bariatric surgery, GLP-1 receptor agonists, or dual GLP-1/GIP agonists and reporting metabolic, inflammatory, or ECM-related outcomes.
3. Systematic reviews and meta-analyses addressing MMPs in obesity or post-treatment conditions.

Exclusion criteria:

1. Case reports, conference abstracts without peer review, narrative commentaries, and non-English publications.
2. Studies not involving obesity or metabolic interventions.
3. Papers with insufficient methodological detail or without measurable data on MMPs or related outcomes.

### 2.3. Screening and Data Extraction

All identified records were screened in a two-stage process:

(1) Title and abstract screening to remove clearly irrelevant publications.
(2) Full-text review of remaining articles to confirm eligibility based on inclusion/exclusion criteria.

Data extraction included study design, sample size, population characteristics, intervention type (surgical or pharmacological), MMPs measured, and principal metabolic or inflammatory outcomes. No automated tools or AI-assisted methods were used during the selection or data extraction process.

### 2.4. Risk of Bias and Quality Assessment

Given the narrative design of this review, no formal quantitative risk-of-bias scoring (e.g., Cochrane or ROBINS-I) was performed. However, methodological quality was qualitatively assessed, taking into account:

- Clarity of study design and inclusion criteria;
- Presence of control or comparison groups;
- Appropriate measurement of MMPs and inflammatory markers;
- Sample size adequacy.

Priority was given to peer-reviewed studies with transparent methodology, and findings from high-quality clinical trials, meta-analyses, and mechanistic studies were emphasized throughout the synthesis.

### 2.5. Data Synthesis

Extracted data were narratively synthesized, focusing on common mechanistic patterns and clinical trends linking MMP modulation with obesity treatment outcomes. Quantitative meta-analysis was not feasible due to heterogeneity in study designs, populations, and outcome measures.

## 3. Matrix Metalloproteinases in Obesity

The complex role of MMPs in obesity reveals their significant impact on ECM remodeling, inflammation, and metabolic disorders. Investigating the function of MMPs provides a foundation for understanding obesity-associated inflammation and developing therapeutic strategies targeting ECM dynamics. This section clarifies how MMP activity differs across tissue compartments, assay types, and disease stages, to reconcile apparent inconsistencies in the literature.

### 3.1. Role and Function of MMPs

MMPs are zinc-dependent endopeptidases that play a central role in ECM remodeling, tissue homeostasis, angiogenesis, and immune regulation. Dysregulated MMP activity contributes to pathological remodeling of adipose tissue, leading to fibrosis, immune-cell infiltration, and impaired adipocyte expansion [8,10]. These alterations disrupt lipid storage, promote ectopic fat deposition, and sustain low-grade inflammation.

The mechanisms by which MMPs affect ECM remodeling involve degradation of collagen, elastin, and fibronectin. Dysregulated angiogenesis, hypoxia, and inflammatory cytokines further enhance MMP expression, creating a vicious cycle that perpetuates adipose dysfunction [8,9]. To correctly interpret these data, it is essential to distinguish between: (i) biological compartment (adipose Tissue Vs. Circulation), (ii) biological level (gene expression, protein concentration, enzymatic activity), and (iii) assay type (qPCR, ELISA/Luminex, zymography). These parameters are not equivalent and often diverge due to tissue-specific regulation, post-transcriptional mechanisms, and the balance between MMPs and their inhibitors (TIMPs) [14,15].

### 3.2. MMP-9: Tissue-Specific Regulation and Systemic Response

Among MMPs, MMP-9 (gelatinase B) has received particular attention due to its link with inflammation, endothelial dysfunction, and cardiovascular risk. Findings on MMP-9 expression in obesity appear contradictory at first glance; however, these discrepancies largely reflect differences in tissue compartments and assay methods.

In adipose tissue, several studies have reported downregulated MMP-9 mRNA expression in obese individuals, even when protein or enzymatic activity is increased [8,14]. This mismatch may result from post-transcriptional regulation, altered protein stability, and depot-specific biology (visceral vs. subcutaneous fat). In circulation, most studies demonstrate elevated MMP-9 protein and activity levels in obesity, which correlate with systemic inflammation (CRP, IL-6, TNF-α) and endothelial dysfunction [10,16]. Bariatric surgery consistently reduces circulating MMP-9 activity, reflecting improved inflammatory and metabolic status [17]. Nevertheless, long-term follow-ups have shown heterogeneous outcomes depending on procedure type, baseline inflammation, and assay technique [11].

**Clarified interpretation**: *Obesity is associated with increased circulating MMP-9 protein/activity and sometimes reduced adipose MMP-9 mRNA expression. These findings are not contradictory but reflect distinct biological processes: systemic inflammation upregulates circulating enzyme activity, while local transcriptional suppression may represent tissue-specific feedback regulation. The decline in MMP-9 levels after bariatric surgery confirms its role as a sensitive marker of inflammation and ECM remodeling [14,16,17].*

### 3.3. MMP-2

MMP-2 (gelatinase A) contributes to degradation of collagen IV, elastin, and fibronectin, and plays an important role in adipose tissue remodeling and vascular function. Elevated circulating MMP-2 levels are associated with BMI, fasting glucose, and leptin resistance. Mechanistically, MMP-2 cleaves the extracellular domain of the hypothalamic leptin receptor, impairing appetite control and promoting weight gain [1]. After bariatric surgery, normalization of MMP-2 levels and activity has been observed, reflecting reduced inflammation and improved ECM homeostasis [17].

### 3.4. MMP-14 (MT1-MMP): Stage-Dependent Role in Adipose Remodeling

MMP-14 (MT1-MMP) demonstrates dual, stage-dependent activity in obesity. In early obesity, characterized by adipose tissue expansion, limited fibrosis, and mild or absent insulin resistance, MMP-14 facilitates ECM degradation and remodeling, promoting healthy adipocyte hypertrophy and angiogenesis. This adaptive activity helps accommodate energy storage and maintain tissue plasticity [8,9]. In advanced obesity, typically associated with longer disease duration, extensive fibrosis, macrophage infiltration, and overt metabolic dysfunction, persistent MMP-14 overexpression becomes pathogenic. It drives fibrosis, inflammation, and endotrophin production—a collagen VI cleavage product that exacerbates insulin resistance and lipid dysregulation [9,10].

**Clarified interpretation:** *The early stage corresponds to adipose tissue expansion with minimal fibrosis and short disease duration, whereas the advanced stage involves fibrotic, inflamed adipose tissue with increased collagen VI/endotrophin and systemic inflammation [9,10,16]. Thus, MMP-14 acts as a context-dependent modulator—beneficial during adaptive ECM remodeling but deleterious when fibrosis predominates.*

### 3.5. Other MMPs and TIMPs

Other MMPs, including MMP-3, MMP-8, MMP-11, and MMP-12, also contribute to ECM remodeling in obesity. Abdominal white adipose tissue shows increased MMP-3, MMP-11, MMP-12, and MMP-14 and decreased MMP-7 and MMP-9, reflecting depot-specific compensatory mechanisms [8,9]. Following bariatric surgery, MMP-8 levels decline, correlating with improvements in leptin and glycemic control [12]. The imbalance between MMPs and TIMPs promotes excessive ECM degradation, adipose fibrosis, and inflammation, and is considered an index of ECM stability and inflammation progression [15].

### 3.6. Integrative Summary

Overall, dysregulated MMP activity creates a feedback loop that sustains ECM remodeling, inflammation, and metabolic dysfunction in obesity. MMP-2 and MMP-9 mirror systemic inflammation and metabolic stress, whereas MMP-14 defines the local structural fate of adipose tissue—adaptive in early remodeling, fibrogenic in chronic disease. Modulating MMP activity represents a promising approach for controlling both metabolic and structural complications of obesity. A summary of individual MMPs, their biological actions, roles in obesity, and clinical significance is presented in Table 1.

## 4. Obesity-Related Inflammation

Obesity is increasingly recognized as a chronic low-grade inflammatory condition associated with adipose tissue dysfunction, immune cell infiltration, and dysregulated extracellular matrix (ECM) remodeling [2,3,13]. As adipose tissue expands, local hypoxia, oxidative stress, and lipotoxicity activate inflammatory pathways that perpetuate systemic metabolic disturbances [4,9].

### 4.1. Immune Cell Polarization and Adipose Inflammation

During obesity progression, the adipose tissue immune profile shifts from an anti-inflammatory to a pro-inflammatory phenotype. The balance between M2 (anti-inflammatory) and M1 (pro-inflammatory) macrophages becomes disrupted, leading to increased production of cytokines such as tumor necrosis factor-alpha (TNF-α), interleukin-1β (IL-1β), and interleukin-6 (IL-6) [2,4]. These cytokines further recruit macrophages, activate NF-κB signaling, and promote chronic low-grade inflammation that extends beyond adipose tissue to the liver, skeletal muscle, and vasculature [8,9].

Other immune cells also contribute to this pro-inflammatory environment.

Th1 and Th17 lymphocytes, neutrophils, and mast cells are upregulated, while regulatory T cells (Tregs) and eosinophils are reduced, weakening local immunoregulation [8,14]. This persistent inflammatory state triggers continuous ECM remodeling, fibroblast activation, and excessive collagen deposition, leading to tissue fibrosis and impaired adipose plasticity [4,9].

### 4.2. Cytokine–MMP Interactions

Pro-inflammatory cytokines regulate MMP expression and activity in both adipose tissue and the vascular wall.

- TNF-α and IL-1β are potent inducers of MMP-1, MMP-3, and MMP-9, accelerating ECM degradation and inflammatory cell infiltration [2,3].
- IL-6 upregulates MMP-2 and MMP-9 expression, linking inflammation to vascular remodeling and insulin resistance [4].
- Transforming growth factor-beta (TGF-β) exerts a dual effect: it suppresses MMP transcription while enhancing TIMP expression, promoting ECM accumulation and fibrosis [8,9].

Together, these cytokine–MMP interactions form a feedback loop in which cytokine-driven MMP overexpression perpetuates ECM degradation, tissue remodeling, and chronic inflammation [9,15]. The major inflammatory factors influencing MMP expression and their metabolic consequences are summarized in Table 2.

To enhance clarity and avoid repetitive descriptions, these relationships are summarized in Table 3 below.

### 4.3. Integrative Perspective

Chronic low-grade inflammation in obesity maintains a self-reinforcing cycle between cytokine production, MMP activation, and ECM disorganization. Excessive MMP activity, driven by pro-inflammatory cytokines, accelerates tissue remodeling and fibrosis, further impairing adipose tissue plasticity and insulin sensitivity [2,8,9]. Targeting the cytokine–MMP axis could therefore restore ECM homeostasis and mitigate obesity-related metabolic complications.

## 5. Impact of Bariatric Surgery

Bariatric surgery represents one of the most effective interventions for sustained weight reduction and metabolic improvement in obesity. Beyond caloric restriction and hormonal changes, it exerts significant effects on systemic inflammation and ECM remodeling through modulation of MMP activity [16,17].

This review summarizes current evidence on MMP dynamics after different bariatric procedures and highlights key factors contributing to variability in reported outcomes.

### 5.1. General Effects on MMPs and ECM Remodeling

Weight loss achieved after bariatric surgery is associated with favorable changes in inflammatory markers and MMP activity, reflecting partial normalization of ECM metabolism and vascular homeostasis [9,14]. Circulating levels of MMP-9 and MMP-2 typically decline after surgery, coinciding with reductions in CRP, IL-6, and TNF-α [11]. However, these associations do not necessarily imply direct causality; MMP modulation likely results from a combination of reduced adipose inflammation, improved insulin sensitivity, and changes in adipokine signaling rather than from weight loss per se. At the tissue level, decreased MMP-9 and MMP-2 activity after surgery suggests remodeling of adipose ECM towards a less fibrotic phenotype. Enhanced tissue perfusion, reduced hypoxia, and shifts in macrophage polarization may contribute to these effects [8].

Nevertheless, in some studies, no significant postoperative change or even a transient increase in MMP-9 has been observed, reflecting early inflammatory remodeling during rapid fat mass reduction [10].

### 5.2. Sources of Heterogeneity Between Studies

Discrepancies in reported MMP outcomes after bariatric surgery can be largely explained by several methodological and clinical factors:

- Type of surgery—Roux-en-Y gastric bypass (RYGB), sleeve gastrectomy (SG), and adjustable gastric banding (AGB) differ in metabolic and hormonal impact, influencing MMP expression differently. RYGB tends to induce more pronounced decreases in circulating MMP-9 compared to SG or AGB [17].
- Follow-up duration—early studies (<3 months) may capture acute inflammatory remodeling, while long-term analyses (>12 months) reflect stable metabolic adaptation.
- Sample type—tissue-specific data (adipose biopsies) often diverge from plasma or serum results due to compartmental regulation and local ECM turnover [4].
- Assay variability—differences in analytical methods (ELISA, zymography, or gene expression assays) can affect measured MMP values and comparability across studies.

Acknowledging these factors is crucial to interpret heterogeneous findings and identify genuine biological trends rather than methodological artifacts.

### 5.3. MMP–PGE2 Axis: Potential Link and Knowledge Gap

Prostaglandin E2 (PGE2) has been proposed as a potential modulator of MMP expression following bariatric surgery. PGE2 can upregulate MMP-2 and MMP-9 via cyclic AMP (cAMP)–protein kinase A (PKA)-mediated activation of transcription factors such as AP-1 and NF-κB [3]. These pathways may contribute to transient ECM degradation during early postoperative remodeling phases.

However, evidence supporting a direct causal relationship between PGE2 signaling and MMP regulation after bariatric surgery remains limited. Most findings are derived from in vitro or animal models, with few human studies directly assessing this mechanism.

Further research is needed to elucidate the extent to which the PGE2–MMP axis contributes to postoperative ECM remodeling and metabolic recovery.

### 5.4. Integrative Perspective

Overall, bariatric surgery leads to substantial improvements in ECM homeostasis and inflammation, reflected by reduced circulating MMP-9 and MMP-2 activity and normalization of TIMP expression. The magnitude and direction of these effects depend on the surgical technique, follow-up duration, and biological compartment analyzed. Future studies integrating tissue and plasma measurements, combined with standardized follow-up protocols, are necessary to clarify the temporal dynamics of MMP regulation after bariatric surgery. Additionally, mechanistic exploration of lipid mediators such as PGE2 may provide new insights into ECM–metabolic crosstalk during postoperative adaptation.

## 6. Metabolic Outcomes

Bariatric surgery results in profound metabolic improvements that extend beyond weight reduction, encompassing normalization of inflammatory activity, glucose metabolism, and lipid homeostasis. These systemic benefits are closely linked to the restoration of ECM balance and modulation of MMPs activity [4,17].

To enhance clarity, the metabolic effects are summarized below according to major functional categories.

### 6.1. Inflammation

Sustained postoperative weight loss is associated with reductions in circulating inflammatory mediators, including C-reactive protein (CRP), TNF-α, IL-6, and IL-1β [8,9]. These changes parallel a decrease in MMP-2 and MMP-9 activity, suggesting that improved ECM stability contributes to the attenuation of systemic inflammation. In adipose tissue, reduced macrophage infiltration and normalization of the M1/M2 ratio further reinforce anti-inflammatory signaling [14]. However, transient postoperative increases in MMP activity have been described during early tissue remodeling, indicating a short-term inflammatory response before metabolic stabilization [10].

### 6.2. Glycemic Control

Improvements in insulin sensitivity and glucose metabolism following bariatric surgery are among the most consistent outcomes. Normalization of MMP-2 and MMP-9 levels correlates with enhanced insulin signaling and reduced leptin resistance, possibly due to decreased proteolytic cleavage of the leptin receptor [11]. Some evidence suggests that lower MMP activity may help preserve pancreatic β-cell function by mitigating systemic inflammation and oxidative stress [8]. Nevertheless, the mechanistic links between MMP inhibition and glucose homeostasis remain largely associative and require further exploration.

### 6.3. Lipid Profile

Postoperative improvements in lipid metabolism include reduced total cholesterol, LDL, and triglyceride levels, alongside increased HDL concentration [17]. These effects are accompanied by downregulation of MMP-8 and partial normalization of MMP/TIMP balance, indicating restoration of vascular integrity and endothelial function [12]. Because MMPs influence lipoprotein remodeling and vascular wall remodeling, these changes likely contribute to reduced cardiovascular risk after surgery.

### 6.4. Weight Reduction and Body Composition

Substantial and sustained weight loss following bariatric surgery is accompanied by structural reorganization of adipose tissue ECM. Decreased collagen deposition, lower MMP-14 expression, and improved angiogenesis reflect reversal of fibrotic remodeling [4,8]. However, variability in ECM adaptation depends on the magnitude of weight loss, type of surgery, and duration of follow-up. Long-term studies suggest partial reaccumulation of ECM stiffness and MMP activity in patients who experience weight regain or persistent metabolic inflammation. A summary of metabolic outcomes after bariatric surgery and their relationship with MMP regulation is presented in Table 4.

### 6.5. Long-Term Effects and Research Gaps

Despite the well-documented short- and medium-term benefits of bariatric surgery, the long-term (>2 years) impact on MMP activity and ECM remodeling remains poorly understood. Available studies are limited by small sample sizes, heterogeneous methodologies, and inconsistent follow-up durations. While initial MMP suppression is commonly observed, sustained normalization over two years has not been conclusively demonstrated.

Future longitudinal research integrating multi-compartment (plasma and tissue) MMP profiling, standardized surgical comparisons, and metabolomic correlations is essential to clarify the temporal dynamics of MMP regulation after bariatric surgery. Additionally, mechanistic exploration of lipid mediators such as PGE2 may provide new insights into ECM–metabolic crosstalk during postoperative adaptation.

Growing evidence also suggests that circulating MMP-2 and MMP-9 levels could serve as potential biomarkers of low-grade inflammation and cardiovascular risk in obesity. Their dynamic changes during treatment may reflect both vascular remodeling and metabolic improvement. However, the lack of standardized assays and reference ranges currently limits their clinical applicability, emphasizing the need for prospective validation studies to confirm their diagnostic and prognostic value.

## 7. Pharmacological Treatment Options

Until recently, bariatric surgery had been considered the most effective and durable intervention for obesity and its associated metabolic disorders. However, the therapeutic landscape has evolved substantially with the emergence of incretin-based pharmacotherapy, particularly GLP-1 receptor agonists (GLP-1 RAs) and dual GIP/GLP-1 receptor agonists, which achieve weight loss and metabolic improvements that approach the outcomes of surgical interventions in selected patients [5,6].

This review highlights the molecular and metabolic mechanisms by which these pharmacotherapies modulate inflammation, ECM remodeling, and MMP activity, and compares their effects to those observed after bariatric surgery.

### 7.1. GLP-1 Receptor Agonists

GLP-1 receptor agonists (e.g., liraglutide, semaglutide) act primarily by enhancing glucose-dependent insulin secretion, delaying gastric emptying, and promoting satiety through hypothalamic pathways [7]. Beyond metabolic regulation, GLP-1 RAs exert anti-inflammatory and anti-fibrotic effects that indirectly influence ECM remodeling.

Studies have shown that treatment with GLP-1 RAs reduces serum levels of CRP, IL-6, and TNF-α, paralleled by a decline in MMP-2 and MMP-9 concentrations [8,9]. These effects likely arise from the suppression of macrophage infiltration and decreased activation of NF-κB and AP-1 pathways in adipose tissue.

In vitro data also suggest that GLP-1 signaling reduces oxidative stress and endothelial dysfunction by attenuating MMP-mediated degradation of basement membrane components [8].

Consequently, GLP-1 RAs appear to improve ECM integrity while mitigating the chronic low-grade inflammation typical of obesity.

### 7.2. Dual GIP/GLP-1 Receptor Agonists

Dual agonists such as tirzepatide activate both the glucose-dependent insulinotropic peptide (GIP) and GLP-1 receptors, producing synergistic effects on glycemic control and weight reduction [6].

Tirzepatide has demonstrated superior efficacy compared to single GLP-1 RAs, with mean body weight reductions of 20–25% in clinical trials [7]. From a mechanistic standpoint, GIP receptor activation enhances insulin sensitivity and promotes lipid oxidation, while concurrent GLP-1 signaling suppresses appetite and inflammation.

Although data on MMP modulation by dual agonists remain limited, early studies indicate a downregulation of MMP-9 and MMP-14 expression in adipose tissue and vasculature, reflecting reduced ECM degradation and fibrosis [8]. These findings align with the concept that incretin-based pharmacotherapy not only induces weight loss but also restores ECM homeostasis.

### 7.3. Comparative Mechanisms: Pharmacotherapy vs. Bariatric Surgery

Both bariatric surgery and incretin-based pharmacotherapy improve metabolic outcomes through overlapping molecular mechanisms.

Shared pathways include:

- Reduction in systemic inflammation and oxidative stress;
- Restoration of MMP/TIMP balance;
- Suppression of NF-κB–dependent cytokine signaling;
- Improved angiogenesis and ECM remodeling.

However, certain mechanistic differences remain notable.

Bariatric surgery induces rapid hormonal and microbial changes that accelerate metabolic improvement, while pharmacological therapies exert gradual, receptor-mediated effects.

Furthermore, the magnitude of ECM remodeling following GLP-1/GIP treatment may be less pronounced but more physiologically regulated, minimizing postoperative inflammatory surges observed after surgery.

Collectively, these data suggest that incretin-based therapy could mimic several beneficial effects of surgery on ECM and MMP regulation, though long-term data are still needed to confirm the durability of these changes.

### 7.4. Emerging Therapeutic Paradigm

The current management of obesity is shifting from a surgery-centric toward a multimodal, individualized approach that integrates pharmacotherapy, metabolic surgery, and lifestyle modification. GLP-1 and dual GIP/GLP-1 receptor agonists provide a less invasive alternative for patients with moderate obesity or those at high surgical risk, while bariatric surgery remains essential for severe or refractory cases.

Ongoing research explores combination strategies, including pre- or post-surgical incretin therapy, aimed at optimizing ECM remodeling, metabolic balance, and long-term weight maintenance. As these therapeutic modalities converge mechanistically—particularly in the modulation of MMPs and ECM integrity—they represent complementary rather than competing options in the evolving treatment landscape of obesity. A comparative overview of bariatric surgery and incretin-based pharmacotherapy in relation to metabolic and ECM-related outcomes is shown in Table 5.

### 7.5. Research Gaps

Although incretin-based pharmacotherapy has revolutionized the management of obesity, long-term data on MMP activity and ECM dynamics remain scarce. Most available studies report short- to medium-term outcomes, and the persistence of MMP suppression beyond two years has yet to be verified.

Further research should aim to directly compare molecular remodeling patterns between surgical and pharmacological interventions using standardized methodologies, tissue-specific analyses, and extended follow-up durations.

## 8. Limitations and Future Directions

Despite significant advances in understanding the interplay between obesity, ECM remodeling, and MMP regulation, several important limitations and knowledge gaps remain. The available evidence is largely derived from small, heterogeneous cohorts and short-term studies, which restricts the ability to establish causal relationships or to generalize findings across different obesity phenotypes and treatment modalities.

### 8.1. Methodological and Clinical Heterogeneity

Considerable variability exists between studies investigating MMP dynamics in obesity and after treatment. Differences in surgical technique (Roux-en-Y gastric bypass, sleeve gastrectomy, adjustable gastric banding), follow-up duration, and biological sample type (plasma vs. adipose tissue) significantly influence reported outcomes.

Moreover, analytical inconsistencies—such as the use of diverse assays (ELISA, zymography, RT-qPCR)—further complicate comparisons across studies. Future research should implement standardized protocols for MMP quantification, stratified by tissue type, surgical procedure, and clinical context, to improve reproducibility and translational value.

### 8.2. Long-Term Effects of Bariatric Surgery

Although bariatric surgery consistently reduces systemic inflammation and MMP activity in the short term, its long-term (>2 years) effects remain poorly defined. Most studies report early postoperative reductions in circulating MMP-2 and MMP-9, yet data beyond two years are limited and often contradictory. Key unanswered questions include whether these changes persist with stable weight maintenance, or whether MMP activity re-emerges in cases of weight regain or residual inflammation.

Longitudinal, multicenter studies integrating molecular, metabolic, and imaging endpoints are required to clarify the durability of ECM remodeling after surgery.

### 8.3. Mechanistic Insights into GLP-1 and GIP-Based Therapies

Incretin-based pharmacotherapies, including GLP-1 and dual GIP/GLP-1 receptor agonists, represent a transformative advance in obesity management, yet their mechanistic effects on ECM turnover and MMP regulation remain insufficiently understood. Existing studies are predominantly clinical and descriptive, lacking detailed molecular analyses of signaling pathways linking incretin receptor activation to ECM remodeling.

Future research priorities include:

- Controlled mechanistic trials evaluating MMP and TIMP activity during GLP-1 and GIP agonist therapy;
- Integration of molecular biomarkers (e.g., collagen turnover markers, endotrophin) with metabolic outcomes;
- Exploration of potential synergistic effects between pharmacological and surgical interventions on ECM homeostasis.

### 8.4. Integrative Research Priorities

A coordinated research framework should aim to elucidate how distinct therapeutic modalities—surgery, pharmacotherapy, and lifestyle intervention—converge on ECM biology and metabolic remodeling.

Priority areas include:

- Long-term MMP dynamics after bariatric surgery, including tissue-specific and systemic effects.
- Mechanistic elucidation of incretin signaling and its downstream influence on ECM integrity.
- Identification of factors driving heterogeneity across studies, such as surgical procedure, patient phenotype, and assay methodology.
- Development of standardized MMP biomarker panels for clinical monitoring and prediction of treatment response.

Future studies should also explore whether baseline or early postoperative MMP levels can predict the magnitude of metabolic improvement or weight loss following surgery and pharmacotherapy. Establishing MMPs as predictive biomarkers could facilitate personalized treatment strategies and improve long-term outcomes in obesity management.

Only through integrative, multicenter, and mechanistically oriented research can we determine whether modulation of MMP activity represents a unifying pathway mediating the metabolic benefits of obesity treatment.

### 8.5. Summary

While substantial progress has been made in linking MMP activity to metabolic improvement, the field remains at an early translational stage. Future studies should bridge the gap between molecular mechanisms and clinical outcomes by employing multi-omics approaches, tissue-level analyses, and long-term follow-up.

Such efforts will enable the identification of precise therapeutic targets within the ECM–MMP axis and foster the development of personalized interventions that extend beyond weight reduction alone.

## 9. Conclusions

This review highlights the critical role of matrix metalloproteinases (MMPs) in the pathophysiology of obesity and their modulation by both bariatric surgery and incretin-based pharmacotherapy.

MMP-2, MMP-9, and MMP-14 emerge as key mediators linking extracellular matrix (ECM) remodeling, inflammation, and metabolic dysfunction.

Both treatment modalities—surgical and pharmacological—demonstrate significant potential to restore ECM homeostasis, attenuate systemic inflammation, and improve insulin sensitivity through partially overlapping molecular mechanisms.

Despite these promising insights, current evidence remains limited by short follow-up periods, heterogeneity in study designs, and inconsistent methodological approaches. Most studies assess circulating MMPs rather than tissue-specific activity, and long-term (>2 years) data on postoperative or pharmacological outcomes are scarce.

Furthermore, mechanistic links between incretin signaling and ECM remodeling are still largely speculative.

Future research should prioritize longitudinal, mechanistic, and comparative studies that evaluate MMP regulation across different therapeutic strategies, integrating molecular biomarkers, metabolic endpoints, and tissue-level analyses. Establishing standardized assays and unified reporting frameworks will be essential to determine whether modulation of the MMP–ECM axis represents a shared mechanism driving metabolic recovery in obesity.

## Figures and Tables

**Table 1:** Summary of individual MMPs: biological action, role in obesity, and clinical significance.

MMP	Main Biological Action	Effect on Obesity/Pathophysiological Mechanisms	Clinical Significance	Sources
MMP-2	Degradation of collagen IV, elastin, fibronectin; ECM remodeling	- ↑ Elevated in obesity - Associated with BMI, glucose, body weight - Causes leptin resistance by cutting the leptin receptor in the hypothalamus	Therapeutic target—its inhibition improves leptin signaling and insulin resistance	[11,14]
MMP-9	Degradation of collagen IV and gelatinase; macrophage infiltration	- ↑ Elevated in obesity - Correlates with CRP, IL-6, TNF-α - Promotes inflammation and endothelial dysfunction	↓ Decreases after bariatric surgery → reduction in cardiovascular risk	[9,16,17]
MMP-8	Degradation of type I collagen; regulation of inflammatory response	- Decreases after bariatric surgery - Associated with a decrease in leptin - Correlates with improved glycaemia and HbA1c	It may be a marker of early metabolic improvement after surgery.	[12]
MMP-14 (MT1-MMP)	Activation of pro-MMP-2; ECM remodeling; endotrophin production	- In early obesity, it promotes adipocyte expansion (beneficial) - In advanced obesity → fibrosis, endotrophin, insulin resistance	Effect dependent on stage of obesity; potential target for staged therapy	[8]
MMP-3	Regulation of other MMPs; degradation of proteoglycans	- ↑ Increased in abdominal adipose tissue - May promote inflammatory processes	Limited clinical data; further research is required.	[4]
MMP-7	Degradation of proteoglycans and ECM components	- ↓ Reduced in adipose tissue in obesity (compensatory mechanisms)	Potential protective role in ECM	[4]
MMP-11	ECM remodeling and adipocyte differentiation	- ↑ Elevated in the adipose tissue of obese individuals - Contributes to metabolic dysfunction	Early marker of metabolic disorders	[4]
MMP-12	Macrophage elastase; elastin degradation	- ↑ Elevated in adipose tissue - Associated with macrophage infiltration	May worsen adipose tissue fibrosis	[4]
TIMPs (1–4)	Natural MMP inhibitors	- TIMP/MMP imbalance → predominance of ECM degradation	ECM stability and inflammation progression index	[15]

Note: ↑ denotes upregulation/increase; ↓ denotes downregulation/decrease; → indicates causative or resultant relationship (“leads to” or “results in”).

**Table 2:** Inflammatory factors and their effects on MMP expression and metabolic consequences.

Inflammatory Factor	Site of Release	Effect on MMPs	Metabolic/Clinical Effect	Sources
TNF-α	M1 macrophages, adipocytes	↑ MMP-2, ↑ MMP-9	Insulin resistance, ECM degradation, inflammation progression	[3,9]
IL-1β	M1 macrophages	↑ MMP-2, ↑ MMP-9	Adipose tissue fibrosis, impaired insulin signaling	[2]
IL-6	Adipocytes, immune cells	↑ MMP-9	Systemic inflammatory response, ↑ CRP, risk of T2DM	[3]
PGE2	Adipocytes, vascular tissue	↑ MMP-1, ↑ MMP-2	Vasculitis, ↑ risk of atherosclerosis	[16]
CRP	Liver (induced by IL-6)	Correlates with MMP-2 and MMP-9	Marker of inflammation and cardiovascular complications	[13]
M1 macrophages	Infiltration of adipose tissue	Stimulates MMP-2, MMP-9	Progression of insulin resistance, adipocyte dysfunction	[2]
M2 macrophages	Physiological adipose tissue	No stimulation of MMPs	Protective effect, reduction in inflammation—reduced obesity	[2]

Note: ↑ denotes upregulation/increase.

**Table 3:** Summary of cytokine–MMP interactions in obesity.

Cytokine	Main MMP Targets	Direction of Regulation	Functional Consequence
TNF-α	MMP-1, MMP-3, MMP-9	↑	ECM degradation, macrophage infiltration
IL-1β	MMP-9	↑	Endothelial activation, adipose inflammation
IL-6	MMP-2, MMP-9	↑	Insulin resistance, vascular remodeling
TGF-β	MMP-2, MMP-14 (via TIMPs)	↓ (transcription); ↑ (fibrosis)	ECM accumulation, fibrosis

Note: ↑ denotes upregulation/increase; ↓ denotes downregulation/decrease.

**Table 4:** Summary of metabolic outcomes after bariatric surgery and their relationship with MMP regulation.

Metabolic Domain	Observed Change After Surgery	Associated MMP Effect	Mechanistic Notes
Inflammation	↓ CRP, TNF-α, IL-6	↓ MMP-2, ↓ MMP-9	Reduced macrophage infiltration, restored ECM balance
Glycemia	↓ Fasting glucose, ↑ insulin sensitivity	↓ MMP-2, ↓ MMP-9	Decreased leptin receptor cleavage, improved insulin signaling
Lipid profile	↓ LDL, ↓ TG, ↑ HDL	↓ MMP-8, normalization of TIMP	Improved endothelial function and ECM elasticity
Adipose structure	↓ Collagen VI, ↓ fibrosis	↓ MMP-14	Reversal of adipose fibrosis, improved angiogenesis

Note: ↑ denotes upregulation/increase; ↓ denotes downregulation/decrease.

**Table 5:** Comparative overview of bariatric surgery and incretin-based pharmacotherapy in relation to metabolic and ECM-related outcomes.

Parameter	Bariatric Surgery	GLP-1 RA	Dual GIP/GLP-1 RA
Weight loss	25–35%	15–20%	20–25%
Glycemic control	Rapid, sustained	Gradual, strong	Superior synergistic effect
Inflammation	↓ CRP, ↓ IL-6, ↓ TNF-α	↓ CRP, ↓ IL-6	↓ CRP, ↓ IL-6, ↓ MCP-1
MMP activity	↓ MMP-2, ↓ MMP-9, ↓ MMP-14	↓ MMP-2, ↓ MMP-9	↓ MMP-9, ↓ MMP-14
ECM remodeling	Structural reversal of fibrosis	Stabilization and reduced degradation	Restoration of ECM balance
Invasiveness	Surgical	Pharmacological (injectable)	Pharmacological (injectable)
Long-term data	>10 years	Up to 2 years	Emerging evidence

Note: ↓ denotes downregulation/decrease.

## Data Availability

No new data were created or analyzed in this study. Data sharing is not applicable to this article.

## Abbreviations

AGB	Adjustable Gastric Banding
BMI	Body Mass Index
CRP	C-Reactive Protein
ECM	Extracellular Matrix
GLP-1	Glucagon-Like Peptide-1
GIP	Glucose-Dependent Insulinotropic Polypeptide
HDL	High-Density Lipoprotein
IL-6	Interleukin-6
LDL	Low-Density Lipoprotein
MMP	Matrix Metalloproteinase
NF-κB	Nuclear Factor Kappa B
PGE2	Prostaglandin E2
RYGB	Roux-en-Y Gastric Bypass
SG	Sleeve Gastrectomy
TIMP	Tissue Inhibitor of Metalloproteinases
TNF-α	Tumor Necrosis Factor Alpha
